# Changes in Clonal Poplar Leaf Chemistry Caused by Stem Galls Alter Herbivory and Leaf Litter Decomposition

**DOI:** 10.1371/journal.pone.0079994

**Published:** 2013-11-19

**Authors:** Nora Künkler, Roland Brandl, Martin Brändle

**Affiliations:** Department of Ecology - Animal Ecology, Faculty of Biology, Philipps-Universität Marburg, Marburg, Germany; University Copenhagen, Denmark

## Abstract

Gall-inducing insects are highly specialized herbivores that modify the phenotype of their host plants. Beyond the direct manipulation of plant morphology and physiology in the immediate environment of the gall, there is also evidence of plant-mediated effects of gall-inducing insects on other species of the assemblages and ecosystem processes associated with the host plant. We analysed the impact of gall infestation by the aphid *Pemphigus spirothecae* on chemical leaf traits of clonal Lombardy poplars (*Populus nigra* var. italica) and the subsequent effects on intensity of herbivory and decomposition of leaves across five sites. We measured the herbivory of two feeding guilds: leaf-chewing insects that feed on the blade (e.g. caterpillars and sawfly larvae) and skeletonising insects that feed on the mesophyll of the leaves (e.g. larvae of beetles). Galled leaves had higher phenol (35%) and lower nitrogen and cholorophyll contents (35% respectively 37%) than non-galled leaves, and these differences were stronger in August than in June. Total herbivory intensity was 27% higher on galled than on non-galled leaves; damage by leaf chewers was on average 61% higher on gall infested leaves, whereas damage by skeletonising insects was on average 39% higher on non-galled leaves. After nine months the decomposition rate of galled leaf litter was 15% lower than that of non-galled leaf litter presumably because of the lower nitrogen content of the galled leaf litter. This indicated after-life effects of gall infestation on the decomposers. We found no evidence for galling x environment interactions.

## Introduction

By living within plant tissue and inducing tumour-like growth [1], gall-inducing insects modulate the growth and physiology of the attacked organs to the insects’ advantage [2]–[5]. Although interactions of gall-inducing aphids and their host plants have been extensively studied [6]–[8] some issues remain: (i) whether other species associated with the host plant differing in specialization respond differently to the effects of gall-infestation, (ii) whether there are galling x environment interactions and (iii) whether phenotypic plasticity affects gall-mediated interactions.

It is well known that the genotype of plants affects ecological interactions [7], [9]. When using host individuals, which may belong to different genotypes, variations of the effects of galling among sites may be due to effects of the host genotype, effects of the environment and various interactions [9]. Therefore, to analyse the importance of the environment as well as the interaction galling x environment, a single genotype distributed across different environments is required. Clonal plants are ideal model systems for such analyses. Choosing a clonal plant provides also the opportunity to evaluate the importance of phenotypic plasticity on gall-mediated interactions between host plants and associated organisms or ecological processes. In the absence of variation in the genome, heritable differences in gene expressions are well known [10]. Thus, variations in response to gall infestation may not only occur among sites but also among genetically identical individuals within sites.

The Lombardy poplar (*Populus nigra* var. *italica*) is a natural variation of black poplar (*Populus nigra* Linnè), discovered at the beginning of the 18^th^ century in northern Italy and subsequently propagated from cuttings to cultivate as ornamental trees throughout Europe. All individuals planted in Germany and probably in all other parts of Europe belong to one clone [11]. Therefore, bottom-up effects mediated by the plant genotype on higher trophic levels, as reported for poplar hybrids by Dickson and Whitham [6] and Schweitzer et al. [7], can be ruled out. The gall-inducing aphid *Pemphigus spirothecae* Passerini (Pemphigidae) is a specialized species restricted to the Lombardy and black poplars [12]. The aphid induces a conspicuous 2–4 cm spiral gall on the petiole of the leaves. These galls comprise the holocyclic generations of *P. spirothecae* from April to November [12]. A distinct feature of the induced gall chamber is that it stretches exclusively across the petioles. Most previous studies have focused on leaf galls stretching across the leaf lamina and did not separate gall tissue from surrounding tissues before processing chemical analyses or conducting experiments. The placement of the *P. spirothecae* galls on the petioles allows an easy removal of the gall chamber from galled leaves without disturbing the leaf lamina [8]. This facilitates for instance studying the effects of galling on decomposition of the adjacent leaf blade without the confounding effects of the gall chamber [7], [8], [13].

In this study, we firstly analysed the effects of *P. spirothecae* on (i) leaf chemistry (phenolic compounds, carbon-nitrogen ratio, chlorophyll content), (ii) leaf attack by herbivorous insects and (iii) decomposition rate of Lombardy poplar leaves. Based on the results of previous research [6], [7] and assuming that galls are physiological sinks [14] we expect a higher phenol content, a higher carbon-nitrogen ratio and a lower chlorophyll content in galled leaves compared with non-galled leaves. To avoid removal of leaf tissue gall-mediated nutrient interception or manipulation of defence chemistry should reduce the susceptibility of galled leaves to other herbivores [6], [15], [16]. Therefore, galled leaves should suffer less herbivory than non-galled leaves. Gall-modulated changes on living plant compartments may also alter after-life processes in the same direction [7]. Therefore, we expect a lower decomposition of galled leaves compared to non-galled leaves. Secondly we also examined whether these effects are influenced by the environment, in particular environmentally driven interactions (galling x environment interaction) and whether phenotypic plasticity causes variation among Lombardy poplar trees.

## Materials and Methods

### Study sites and sampling

Five study sites with old-growth Lombardy poplar individuals were selected in the surroundings of Marburg (Hesse/Germany) at elevations ranging from 176 m to 246 m a.s.l. At all sites, poplars were planted isolated from other tree species as alley trees with equal spacing. Although we have no data about the age of trees, all trees within sites were approximately at same height suggesting a common date of planting. Land uses of the surrounding areas ranged from intensive agriculture (two sites) to extensive livestock farming (two sites). One site was surrounded by ruderal vegetation. At each site, at least seven poplar trees were present. Five trees per site were chosen for subsequent analyses (total 25 trees). We verified the clonal identity of all considered trees using seven specific microsatellites [17]. Our study sites were neither privately owned nor protected areas. No specific permits were required because no protected or endangered species were sampled.

On three dates in 2009 (June, July and August), we sampled five galled and five non-galled leaves from the lower parts of each tree (up to 2 m). The position of collected leaves pairs within branches thereby varied and depended on the availability of galled leaves. Non-galled leaves were always collected in the immediate vicinity of the infested leaves. We estimated the proportion of the leaf area removed by folivores of 750 sampled leaves (10 leaves per tree×5 trees×5 sites×3 dates) and measured the chemical components of leaves sampled in June and August (500 leaves).

### Leaf chemistry and herbivory

To obtain sufficient tissue for the chemical analyses, galled and non-galled leaves from each tree and sampling date were pooled separately. We estimated the total phenolic content of leaves collected in June and August (petioles and hence gall chambers removed) using a modification of the Folin–Ciocalteu method [18] and determined the chlorophyll concentration according to Holden [19]. We analysed the carbon and nitrogen contents using an Elementar Vario EL element analyser (Elementar Analysengeräte Hanau). Damage caused by folivores was categorized into two feeding types: leaf chewers, which feed from the leaf edges (e.g. caterpillars and sawfly larvae), and skeletonisers, which feed on the leaf mesophyll (e.g. larvae of leaf beetles). One of us visually estimated the percentage of damage to the galled and non-galled leaves caused by each feeding type, as well as the total leaf area removed (including damage caused by additional folivorous insects not belonging to these two groups) at levels of 2%, 5% and further upwards in 5% steps with the help of pictures presenting different levels of damage.

### Decomposition experiment

In October 2009, we collected at each study site freshly fallen galled and non-galled leaves from the ground. For the decomposition experiment, air-dried galled and non-galled leaf litter were placed separately into litter bags (4 g±0.1 g). From both the non-galled and the gall-infested leaves we previously removed the petioles and therefore on gall-infested leaves the gall chambers. Each category (galled *versus* non-galled) was replicated 10 times per site (20 litter bags per site). Litter bags were of coarse mesh size (5 mm × 5mm) to allow passage of soil macro- and meso-invertebrates. In November 2009, the litter bags were placed in a randomised block design (five blocks, each about 3 m apart) in a “common garden”. We selected a wooded site with oak, larch, birch and beech near our Biology Department building. Half of the litter bags were collected after six months (May 2010) the other half after nine months (August 2010). The total number of litter bags was 100 (5 blocks × 5 sample sites × 2 categories × 2 sampling dates). All samples were air dried, dirt was removed carefully, and the remaining leaf litter was weighted. We also measured the C/N ratio of additional galled and non-galled leaf litter collected in September 2009. At least five samples each of galled and non-galled leaves were taken from every site. To reach the mass necessary for the element analyser, two leaves from each category were pooled.

### Statistical analyses

Prior to all statistical analyses, the proportion of leaf tissue removed by herbivores was arcsine-square-root-transformed to approximate normal distribution of residuals and to reduce variance heterogeneity. Herbivory intensity was averaged across the five single values for each tree. Data were analysed in R with mixed effect models as implemented in the lme4 package [20]. Site, trees within site, and in the leaf-litter experiment “block” were considered as random factors, and galling and sampling data (time) were considered as fixed factors. To test for site effects and whether the strength of the effect of galling varied among sites (environment x galling interaction) and among trees within sites (trees within sites x galling interaction), we compared the likelihood of the full model (including the interaction term) with the likelihood of the model without the effects of “site” or the interaction term respectively via a log-likelihood ratio test (LRT, Edinburgh Psychology R-users: http://psy-ed.wikidot.com/glmm, accessed 2013; for models see Table S5). Significance of fixed factors was approximated using the cftest function in the multcomp package [21]. Relationships between herbivory intensity and C/N ratio as well as between herbivory intensity and phenol content were analysed using linear regression.

## Results

### Leaf chemistry

**Figure 1:**
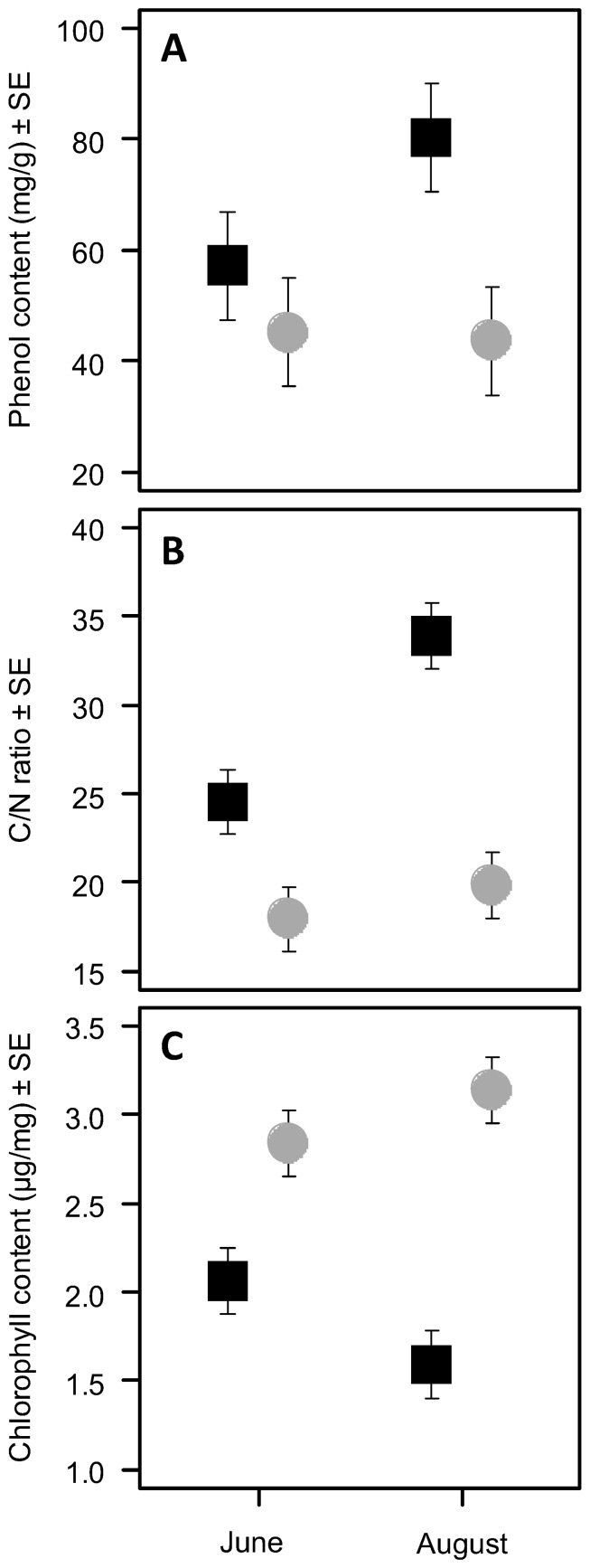
Phenol content, C/N ratio and chlorophyll content of galled (filled black quadrats) and non-galled (filled grey circles) leaves. (A) Average phenol content of galled and non-galled leaves in June and August. (B) Average C/N ratio of galled and non-galled leaves in June and August. (C) Average chlorophyll content measured as µg/mg air-tried leaf mass of galled and non-galled leaves in June and August. Error bars indicate ± SE, n = 100. Values from all trees and sites on the sampling dates indicated were averaged.

Across all trees, sites and sampling dates the leaf blades of galled leaves had a 35 % higher content of phenolic compounds, a 35 % higher C/N ratio and a 37 % lower chlorophyll content relative to non-galled leaves (all P < 0.001, Table 1, Fig. 1). In galled leaves the phenol content and the C/N ratio increased from June to August (Fig. 1A, B). In non-galled leaves we found no such differences (Fig. 1A, B, Table 1). The chlorophyll content of galled leaves decreased from June to August but showed the opposite trend in non-galled leaves (Fig. 1C, Table 1).

**Table 1:** Effects of galling and sampling date and their interaction on phenol content, C/N ratio and total chlorophyll content of Lombardy poplars using a mixed-model approach.

Source	Phenol content (mg/g)				C/N ratio (%)				Total chlorophyll content (µg/mg)			
	Estimate	SE	Z	P	Estimate	SE	z	P	Estimate	SE	z	P
Intercept	43.666	5.813	7.512	**< 0.001**	19.848	0.926	21.425	**< 0.001**	3.139	0.137	22.959	**< 0.001**
Gall [G]	36.641	7.215	5.078	**< 0.001**	14.037	1.300	10.796	**< 0.001**	−1.545	0.135	−11.426	**< 0.001**
Time [T]	1.521	6.888	0.221	0.83	−1.886	1.300	−1.451	0.147	–0.300	0.135	−2.221	**0.026**
G*T	−24.562	9.742	−2.521	**0.012**	−7.452	1.839	–4.053	**< 0.001**	0.771	0.191	4.03	**< 0.001**

Note that *sites* and *tree* were considered as random variables. Significant effects are in bold. n = 100.

### Herbivory

**Figure 2:**
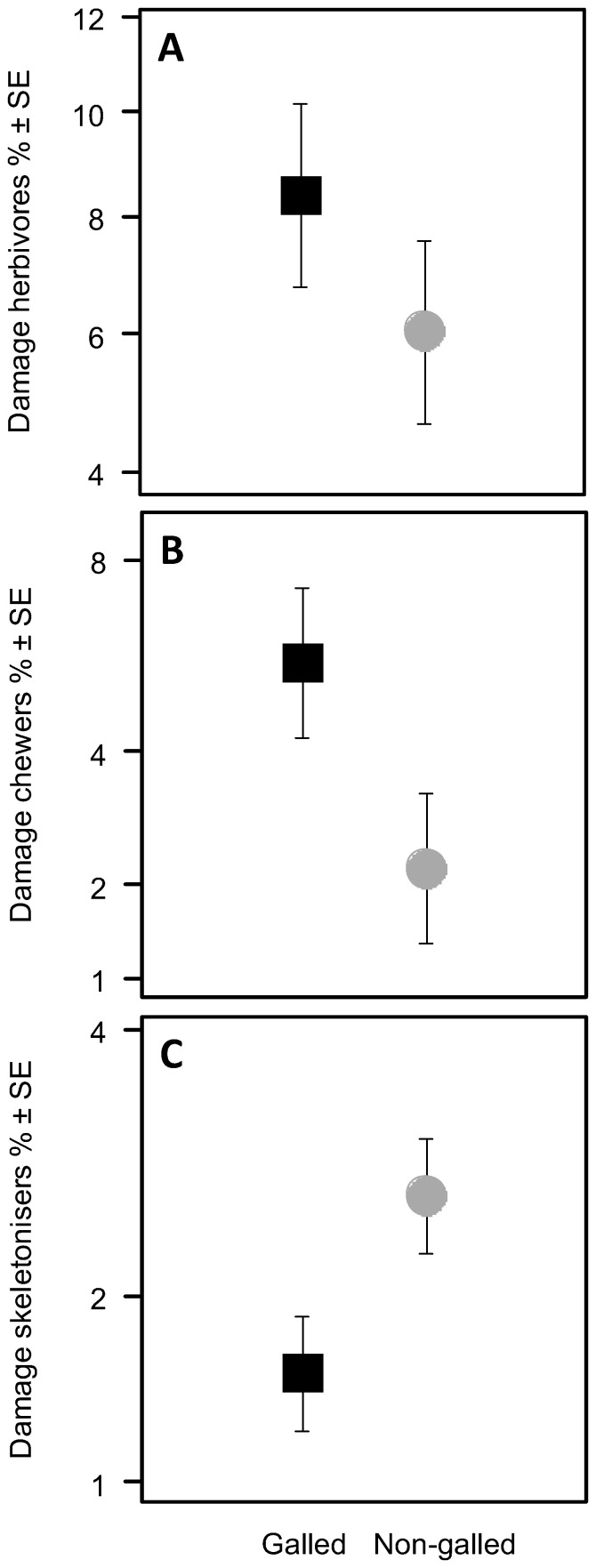
Average total leaf damage caused by herbivores. Leaf damage of galled (filled black quadrats) and non-galled leaves (filled grey circles). Note that y- axes are arcsine-square-root transformed. (A) Total herbivory, (B) herbivory by leaf chewers, (C) herbivory by leaf skeletonisers. Values are averaged across trees, sites and sampling dates. Error bars indicate ± SE, n = 150.

Leaves with galls showed 27% higher loss of material on leaf blades relative to non-galled leaves (Fig. 2A, Table 2). Damage caused by leaf chewers was 61% higher on galled relative to non-galled leaves (Fig. 2B, Table 2), while damage caused by skeletonising insects was 39% lower on galled leaves relative to non-galled leaves (Fig. 2B, Table 2). Since the effects of skeletonising insects could be underestimated because their feeding tracks could in part be removed by leaf chewers, we analysed the effects of skeletonising insects excluding all leaves showing damage caused by leaf chewers (Table S1). The herbivory intensity of skeletonising insects was still higher on non-galled leaves. Linear regression between phenol content and folivore damage revealed negative relationships between phenol content and total herbivory as well as between phenol content and leaf chewer on galled leaves (total herbivory: r = –0.30, P < 0.05, chewers: r = –031, P < 0.05; Table S2). Linear regression between C/N ratio and damage by folivores did not reveal any significant relationship between herbivory intensity within the feeding categories and the C/N ratio (Table S2).

**Table 2:** Effects of galling and sampling date and their interaction on total herbivory, leaf-chewing herbivory and skeletonising herbivory of Lombardy poplars using a mixed-model approach.

Source	Total herbivory				Leaf-chewing herbivory					Skeletonising herbivory				
	Estimate	SE	z	P		Estimate	SE	z	P		Estimate	SE	z	P
Intercept	0.295	0.022	13.555	**<0.001**		0.255	0.024	10.81	**<0.001**		0.107	0.011	9.34	**<0.001**
Gall [G]	−0.086	0.03	−2.83	**0.005**		−0.125	0.032	−3.854	**<0.001**		0.041	0.013	3.21	**0.001**
Time [July]	−0.009	0.03	−0.287	0.774		−0.037	0.032	−1.15	0.250		0.019	0.013	1.512	0.131
Time [August]	0.007	0.03	0.225	0.822		−0.006	0.032	−0.184	0.854		0.0333	0.013	2.618	**0.009**
G*Time [July]	0.038	0.043	0.883	0.377		0.043	0.046	0.931	0.352		−0.013	0.018	−0.74	0.460
G*Time [August]	0.082	0.043	1.906	0.057		0.056	0.046	1.221	0.222		0.009	0.018	0.497	0.619

Note that *sites* and *tree* were considered as random variables. Significant effects are in bold. n = 150.

### Decomposition

**Figure 3:**
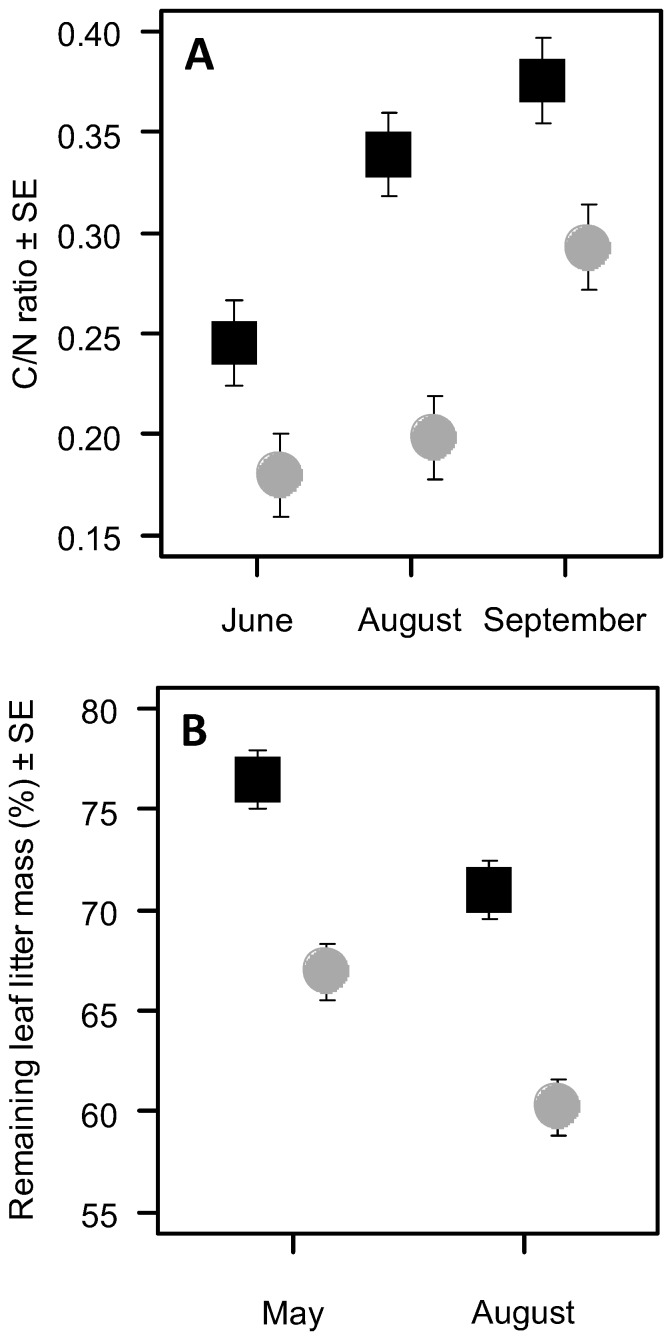
C/N ratio and decomposition of galled and non-galled leaves. (A) C/N ratio of galled (filled black quadrats) and non-galled leaves (filled grey circles) on the three sampling dates. Note that in June and August the C/N ratio was estimated from living leave tissue whereas in September leaf litter was analysed. (B) The average of remaining leaf litter mass of galled and non-galled leaves after six (May) and nine months (August) of decomposition compared to the leaf litter mass on the collection date in November. Values are averages across blocks and sites. Error bars indicate ± SE.

On average, the C/N ratio of tissue of galled leaf litter sampled in September was higher than that of non-galled leaf litter and significantly higher than the values from June and August (Fig. 3A, Table S3). Galled leaf litter decomposed more slowly than non-galled leaf litter (Fig. 3B, Table S4). After six months (May) galling decelerated leaf mass loss by 13% and after nine months (August) by 15% relative to non-galled litter.

### Variation among sites, trees within sites and environment x galling interactions

Chemical leaf contents, herbivory and decomposition of leaves did not differ among sites (all LTR tests P>0.05, Table S5). The effects of galling on the chemical content, leaf herbivory and decomposability of leaves did not change among sites as well as among trees within sites (all LTR tests P>0.05, Table S5).

## Discussion

Galling insects are able to manipulate the growth and development of plant tissue [22] and these insects also modify the nutritional quality and secondary metabolites of host organs [23]–[25]. Several studies have analysed the mechanisms of manipulation and alteration of host plant traits by galling insects [4], [5], [26], [27]. Our study supports previous findings that gall-inducing insects change the chemical composition of surrounding leaf tissue. We found evidence that galling insects indirectly influence the herbivory of other herbivores. But gall-mediated changes in leaf chemistry and interactions with other herbivores were only little affected by galling x environment interactions and phenotypic plasticity.

Galling insects do not only modify the growth of plant tissues, but also the allocation of assimilated compounds and nutrients through the host plant [28], [29]. Leaf galls act as strong physiological sinks [4], [14] as they interrupt the flow of nutrients towards the leaf lamina. We provide evidence that *P. spirothecae* manipulates its host beyond the immediate environment of the gall, as we measured increased phenol and C/N but lower chlorophyll content in galled than in non-galled leaf tissue. This suggests that gall-induced allocation of assimilates are restricted to the galled leaves.

Many plant species are known to respond to galls with local defence, such as the production of secondary metabolites [28]. These reactions might even lead to the death of the galling insects [30]. Contrary to suggestions that elevated amounts of phenolics act as a defence mechanisms against galling insects [31], [32], other studies have shown no relationship between phenol content and plant resistance against galling insects [33], [34]. Galling insects may even benefit from induction of defence metabolites [18]: with increasing defence metabolites, susceptibility to herbivory by other herbivores and therefore potential competitors decreases [19]. But note that in our model system the higher phenol content of galled leaves even increased herbivory of the leaf chewers (see below). Overall, these results suggest that the role of phenolic compounds for plant defence is complex and deserves further detailed studies.

The availability of nitrogen in the host plant organs is essential to the survival of the larvae of the gall former [2]. Galls induced by species of the superfamily Aphidoidea are phloem suckers and act as nitrogen sinks as they directly tap the host vascular system that distributes assimilates and nitrogen compounds [35]. Apparently, the galls of *P. spirothecae* impede nutrient supply of adjacent leaves as we found a lower relative nitrogen content (higher C:N ratio) in galled leaves. Measurements of the chlorophyll content indicated that senescence started earlier in galled leaves than in non-galled leaves. Early leaf senescence may be viewed as a defence strategy of plants against galling. At least for aphid species that form galls on the leaf lamina leaf abscission leads to a high mortality of gall aphids [36], [37]. In our study, however, we did not observe gall abortion caused by induced senescence. Accepting that galls are physiological sinks [17], [38], we suggest that for our model system, a lack of nutrients is more likely responsible for the strong decrease in chlorophyll content and the increase in phenol content in galled leaves than a direct manipulation of secondary metabolites or induction of plant defence by the gall maker. Nitrogen uptake by the galling insect may cause a deficit of nutrients in adjacent leaf blades [2]. A lower nitrogen content in leaf blades in turn may result in a lower photosynthetic capacity as nitrogen is crucial for the photosynthetic pathway [39], [40]. Consequently, the chlorophyll content decreases in galled leaves. However one should note that a strong coupling of N-content and photosynthetic capacity provides probably a too simplified view. Nitrogen could be stored in secondary compounds or other chemical forms which may not be available for photosynthesis.

Nutrient interception or defensive manipulation by gall formers could reduce the susceptibility of galled leaves to herbivores [6], [15], [16]. Leaf galls of *P. spirothecae* cause chemical differences in poplar foliage that may directly decrease the herbivory by folivorous insects. We hypothesized that nutrient interception, active transport of photoassimilates and the changed concentration of secondary defence metabolites render the leaf generally less susceptible to free-feeding herbivores. Our results, however, suggest a much more complex relationship between levels of secondary compounds, nitrogen availability and levels of herbivory of free-feeding insects: Certain types of herbivory even increased with lower N- content and higher content of phenols. Compensatory feeding by adjusting the feeding rate to levels of nitrogen has been found repeatedly and is probably a general phenomenon among herbivorous insects [41]. We therefore suggest that leaf chewers compensate for suboptimal food supply by increasing food intake [30]. In contrast, skeletonising insects feeding on the leaf mesophyll showed a lower level of herbivory on galled leaves than on non-galled leaves. Some insects are known for the strategy of leaving a nutritionally poor plant and searching for an alternative food resource [41]–[43]. The skeletonising insects in our model system may simply switch to the nutrient rich non-galled leaves in the immediate neighbourhood of the galled leaves. Insects specialised on mesophyll feeding may mainly suffer from two classes of phenolic compounds, flavonoids and tannins, as these are stored in plant cell vacuoles and in the sap of living cells [30]. These two common phenolic compounds are known to act as feeding deterrents and hence affect the nutritional value of plant tissue [30], [37].

Plant-mediated interactions also occur indirectly between above-ground herbivores and decomposers [44]. Thereby variations in decomposition rates may be a consequence of differences in leaf chemistry [45]. Foliar herbivory can exert a variety of effects on decomposition processes by influencing the quality of leaves [44], [46], [47]. Altered chemical composition and hence nutrient quality can decrease decomposition rates [46], [48]. Pemphigidae on cottonwood are known to reduce leaf litter quality and decomposition rate [7]. Our decomposition experiment results support these previous findings [8].

The fact that we did not find a difference in response among individual phenotypes suggested that the clonal planting of Lombardy poplars limits the variability of the response of the individual to galling. Therefore, it would be interesting to compare the response of different naturally occurring black popular genotypes. Owing to their sexual reproduction, natural stands of black poplars possess a high intraspecific genetic diversity that facilitates the selection of genotypes with adapted responses to herbivory.

The low variability induced by galling x environment interactions may be explained by several factors. First, among the considered sites, the environmental conditions may have differed insufficiently for affecting the interactions among the gall formers and the host plant. Unfortunately we have at the moment no data about nutrient availability of soils or soil-pH. Second, environment induced changes of interactions may rather causes changes in gall abundance than changes in leaf chemistry [9]. It has been shown that higher nutrient content of plants due to fertilisation increases gall density [9]. Gall induction in turn increases the number of lateral shoots and number of leaves per shoot [49]. Increased foliar abundance in turn increases abundance and diversity of herbivores and their predators. Hence judging the importance of environment on gall-induced interactions requires more detailed field analyses ideally attended by experimental approaches.

## Conclusions

Galls are formed entirely from plant tissue, but the formation and maintenance of galls are controlled by the gall former, thus representing a good example for the concept of the extended phenotype [50]–[53]. Our study demonstrated direct and indirect effects of gall infestation [54]. *P. spirothecae* influences host leaf chemistry, which in turn influences other community members of two trophic levels (other herbivores and decomposers). In our study these interactions were not affected by environmental heterogeneity and phenotypic plasticity. This suggests that the response of poplars is fixed, allowing no or only limited flexibility of phenotypes. Such a fixed response may be caused by the close coevolutionary history of gall formers and their host plant. For the plant, clonal cultivation of poplars may be viewed as a trap or dead end that permits the evolution of protection mechanisms to dispose the parasites. However, controlled experiments are needed varying nutrient supply and other environmental conditions (e.g. water and soil availability) to prove our findings.

## Supporting Information

Table S1 **Effects of galling and sampling date and their interactions on skeletonising herbivory using a mixed-model approach.** All leaves showing herbivory by chewing were excluded from the analysis. Note that *sites* and *tree* were considered as random variables. Significant effects are in bold. To increase sample size, herbivory intensity estimates of single leaves were analysed. n = 369.
(DOCX)

Table S2 **Linear regressions between herbivory intensity, C/N ratio and phenol content (in mg/g -dried leaf mass) calculated separately for galled and non-galled leaves.** Coefficients of determination (R^2^) are shown; significant relationships are in bold (p< 0.05). Signs indicate the direction of the relationship.
(DOCX)

Table S3 **Effects of galling and sample date and their interactions on C/N values (June, August, September) using a mixed-model approach.** Note that *site* was considered as random variable. Significant effects are in bold. n = 149. d.
(DOCX)

Table S4 **Effects of galling, sample date and their interactions on remaining leaf litter mass (mixed-model ANOVA).** Note that *sites* and *block* were considered as random variables. Significant effects are in bold.
(DOCX)

Table S5 **Log-likelihood ratio tests (LRT) for the effect of site, environment x galling and galling among trees within sites on leaf chemistry, herbivory and decomposition.**
(DOCX)
