# Supplementary material for: Changes in Clonal Poplar Leaf Chemistry Caused by Stem Galls Alter Herbivory and Leaf Litter Decomposition
Source: PLoS One. 2013 Nov 19;8(11):e79994. doi: 10.1371/journal.pone.0079994 (PMC3833850; doi:10.1371/journal.pone.0079994)
Supplement: Table S1 — Effects of galling and sampling date and their interactions on skeletonising herbivory using a mixed-model approach. All leaves showing herbivory by chewing were excluded from the analysis. Note that sites and tree were considered as random variables. Significant effects are in bold. To increase sample size, herbivory intensity estimates of single leaves were analysed. n = 369. (DOCX) [file pone.0079994.s001.docx]

Table S1. Effects of galling and sampling date and their interactions on skeletonising herbivory using a mixed-model approach. All leaves showing herbivory by chewing were excluded from the analysis. Note that *sites* and *tree* were considered as random variables. Significant effects are in bold. To increase sample size, herbivory intensity estimates of single leaves were analysed. n = 369.

| Source | Skeletonising herbivory (chewing excluded) | | | |
| --- | --- | --- | --- | --- |
|  | Estimate | SE | Z | P |
| **Intercept** | 0.110 | 0.015 | 7.233 | **< 0.001** |
| **Gall [G]** | 0.073 | 0.018 | 4.148 | **< 0.001** |
| Time [July] | −0.001 | 0.018 | −0.053 | 0.958 |
| Time[August] | −0.035 | 0.018 | −1.918 | 0.055 |
| G*Time[July] | −0.053 | 0.024 | −2.239 | **0.025** |
| G*Time [August] | −0.027 | 0.024 | −1.124 | 0.261 |
