# Supplementary material for: Changes in Clonal Poplar Leaf Chemistry Caused by Stem Galls Alter Herbivory and Leaf Litter Decomposition
Source: PLoS One. 2013 Nov 19;8(11):e79994. doi: 10.1371/journal.pone.0079994 (PMC3833850; doi:10.1371/journal.pone.0079994)
Supplement: Table S2 — Linear regressions between herbivory intensity, C/N ratio and phenol content (in mg/g -dried leaf mass) calculated separately for galled and non-galled leaves. Coefficients of determination (R2) are shown; significant relationships are in bold (p< 0.05). Signs indicate the direction of the relationship. (DOCX) [file pone.0079994.s002.docx]

| Table S2. Linear regressions between herbivory intensity, C/N ratio and phenol content (in mg/g -dried leaf mass) calculated separately for galled and non-galled leaves. Coefficients of determination (R²) are shown; significant relationships are in bold (p< 0.05). Signs indicate the direction of the relationship. | | | | |
| --- | --- | --- | --- | --- |
|  | C/N ratio (%) | | Phenol content (mg/g) | |
|  | Galled | Ungalled | Galled | Ungalled |
| Total herbivory | 0.011 (-) | 0.021 (+) | **0.092 (-)** | 0.055 (-) |
| Chewing | 0.012 (-) | 0.003 (+) | **0.101 (-)** | 0.016 (-) |
| Skeletonising | 0.005 (-) | < 0.001 (-) | 0.003 (-) | 0.073 (+) |
