# Supplementary material for: Changes in Clonal Poplar Leaf Chemistry Caused by Stem Galls Alter Herbivory and Leaf Litter Decomposition
Source: PLoS One. 2013 Nov 19;8(11):e79994. doi: 10.1371/journal.pone.0079994 (PMC3833850; doi:10.1371/journal.pone.0079994)
Supplement: Table S3 — Effects of galling and sample date and their interactions on C/N values (June, August, September) using a mixed-model approach. Note that site was considered as random variable. Significant effects are in bold. n = 149. d. (DOCX) [file pone.0079994.s003.docx]

Table S3. Effects of galling and sample date and their interactions on C/N values (June, August, September) using a mixed-model approach. Note that *site* was considered as random variable. Significant effects are in bold. n = 149.

| Source | C/N | | | |
| --- | --- | --- | --- | --- |
|  | Estimate | SE | Z | P |
| Intercept | 0.198 | 0.010 | 19.751 | **< 0.001** |
| **Gall [G]** | 0.140 | 0.015 | 9.570 | **< 0.001** |
| Time [August] | −0.019 | 0.015 | −1.286 | 0.198 |
| Time[September] | 0.095 | 0.015 | 6.441 | **< 0.001** |
| G*Time [August] | −0.074 | 0.021 | −3.593 | **< 0.001** |
| G*Time [September] | −0.058 | 0.021 | −2.762 | **0.006** |
