# Supplementary material for: Changes in Clonal Poplar Leaf Chemistry Caused by Stem Galls Alter Herbivory and Leaf Litter Decomposition
Source: PLoS One. 2013 Nov 19;8(11):e79994. doi: 10.1371/journal.pone.0079994 (PMC3833850; doi:10.1371/journal.pone.0079994)
Supplement: Table S4 — Effects of galling, sample date and their interactions on remaining leaf litter mass (mixed-model ANOVA). Note that sites and block were considered as random variables. Significant effects are in bold. (DOCX) [file pone.0079994.s004.docx]

Table S4. Effects of galling, sample date and their interactions on remaining leaf litter mass (mixed-model ANOVA). Note that *sites* and *block* were considered as random variables. Significant effects are in bold.

| Source | Remaining leaf litter mass (%) | | | |
| --- | --- | --- | --- | --- |
|  | Estimate | SE | z | P |
| Intercept | 76.562 | 2.499 | 30.634 | **< 0.001** |
| **Gall [G]** | −9.630 | 1.037 | −9.29 | **< 0.001** |
| **Time [T]** | −5.766 | 1.049 | −5.499 | **< 0.001** |
| G*T | −1.139 | 1.473 | −0.773 | 0.439 |
