# Supplementary material for: Changes in Clonal Poplar Leaf Chemistry Caused by Stem Galls Alter Herbivory and Leaf Litter Decomposition
Source: PLoS One. 2013 Nov 19;8(11):e79994. doi: 10.1371/journal.pone.0079994 (PMC3833850; doi:10.1371/journal.pone.0079994)
Supplement: Table S5 — Log-likelihood ratio tests (LRT) for the effect of site, environment x galling and galling among trees within sites on leaf chemistry, herbivory and decomposition. (DOCX) [file pone.0079994.s005.docx]

Table S5. Log-likelihood ratio tests (LRT) for the effect of site, environment x galling and galling among trees within sites on leaf chemistry, herbivory and decomposition.

**Site effects**

Phenol content

model1: phenols~(1|site/tree)+(gall|site/tree)+gall+time+gall*time

model2: phenols~(1|site)+ (1|site/tree)+(gall|site/tree)+gall+time+gall*time

LRT

|  | Df | AIC | BIC | logLik | Chisq | Chi Df | Pr(>Chisq) |
| --- | --- | --- | --- | --- | --- | --- | --- |
| model1 | 13 | 950.87 | 984.74 | -462.44 |  |  |  |
| model2 | 14 | 952.87 | 989.34 | -462.44 | 0 | 1 | 1 |

Chlorophyll content

model1: chlorophyll~(1|site/tree)+(gall|site/tree)+gall+time+gall*time

model2: chlorophyll~(1|site)+ (1|site/tree)+(gall|site/tree)+gall+time+gall*time

LRT

|  | Df | AIC | BIC | logLik | Chisq | Chi Df | Pr(>Chisq) |
| --- | --- | --- | --- | --- | --- | --- | --- |
| model1 | 13 | 169.1 | 202.97 | -71.551 |  |  |  |
| model2 | 14 | 171.1 | 207.57 | -71.55 | 0.001 | 1 | 0.975 |

CN

model1: CN~(1|site/tree)+(gall|site/tree)+gall+time+gall*time

model2: CN~(1|site)+ (1|site/tree)+(gall|site/tree)+gall+time+gall*time

LRT

|  | Df | AIC | BIC | logLik | Chisq | Chi Df | Pr(>Chisq) |
| --- | --- | --- | --- | --- | --- | --- | --- |
| model1 | 13 | 610.16 | 644.03 | -292.08 |  |  |  |
| model2 | 14 | 612.16 | 648.63 | -292.08 | 5.00E-04 | 1 | 0.9824 |

Total herbivory

model1: total_herbivory~(1|site/tree)+(gall|site/tree)+gall+time+gall*time

model2: total_herbivory~(1|site)+ (1|site/tree)+(gall|site/tree)+gall+time+gall*time

LRT

|  | Df | AIC | BIC | logLik | Chisq | Chi Df | Pr(>Chisq) |
| --- | --- | --- | --- | --- | --- | --- | --- |
| model1 | 15 | -216.69 | -171.53 | 123.35 |  |  |  |
| model2 | 16 | -214.69 | -166.52 | 123.35 | 0 | 1 | 0.9999 |

Herbivory chewers

model1: chewers~(1|site/tree)+(gall|site/tree)+gall+time+gall*time

model2: chewers~(1|site)+ (1|site/tree)+(gall|site/tree)+gall+time+gall*time

LRT

|  | Df | AIC | BIC | logLik | Chisq | Chi Df | Pr(>Chisq) |
| --- | --- | --- | --- | --- | --- | --- | --- |
| model1 | 15 | -193.98 | -148.82 | 111.99 |  |  |  |
| model2 | 16 | -191.98 | -143.81 | 111.99 | 0 | 1 | 1 |

Herbivory sceletonisers

model1: sceletonisers~(1|site/tree)+(gall|site/tree)+gall+time+gall*time

model2: sceletonisers~(1|site)+ (1|site/tree)+(gall|site/tree)+gall+time+gall*time

LRT

|  | Df | AIC | BIC | logLik | Chisq | Chi Df | Pr(>Chisq) |
| --- | --- | --- | --- | --- | --- | --- | --- |
| model1 | 15 | -454.47 | -409.31 | 242.23 |  |  |  |
| model2 | 16 | -452.47 | -404.3 | 242.23 | 0 | 1 | 1 |

Decomposition

model1: remaining.leaf.litter.mass.. ~ (1 | block) + (gall| site) + gall + time + gall * time

model2: remaining.leaf.litter.mass.. ~ (1 | site) + (1 | block) + (gall | site) + gall + site + gall * time

LRT

|  | Df | AIC | BIC | logLik | Chisq | Chi Df | Pr(>Chisq) |
| --- | --- | --- | --- | --- | --- | --- | --- |
| model1 | 9 | 562.25 | 585.42 | -272.12 |  |  |  |
| model2 | 10 | 564.25 | 589.99 | -272.12 | 0.0001 | 1 | 0.9938 |

**Galling x environment interaction**

Phenol content

model1: phenols~(1|site/tree)+gall+time+gall*time

model2: phenols~(1|site/tree)+(gall|site)+gall+time+gall*time

LRT

|  | Df | AIC | BIC | logLik | Chisq | Chi Df | Pr(>Chisq) |
| --- | --- | --- | --- | --- | --- | --- | --- |
| model1 | 7 | 939.4 | 957.63 | -462.7 |  |  |  |
| model2 | 10 | 944.87 | 970.92 | -462.44 | 0.523 | 3 | 0.9138 |

Chlorophyll content

model1: chlorophyll~(1|site/tree)+gall+time+gall*time

model2: chlorophyll~(1|site/tree)+(gall|site)+gall+time+gall*time

LRT

|  | Df | AIC | BIC | logLik | Chisq | Chi Df | Pr(>Chisq) |
| --- | --- | --- | --- | --- | --- | --- | --- |
| model1 | 7 | 160.19 | 178.42 | -73.094 |  |  |  |
| model2 | 10 | 163.1 | 189.15 | -71.551 | 3.09 | 3 | 0.3785 |

CN

model1: CN ~ (1 | site/tree) + gall + time + gall * time

model2: CN ~ (1 | site/tree) + (gall | site) + gall + time+ gall * date

LRT

|  | Df | AIC | BIC | logLik | Chisq | Chi Df | Pr(>Chisq) |
| --- | --- | --- | --- | --- | --- | --- | --- |
| model1 | 7 | 600.26 | 618.5 | -293.13 |  |  |  |
| model2 | 10 | 606 | 632.05 | -293 | 0.2673 | 3 | 0.9661 |

Total herbivory

model1: total_herbivory ~ (1 | site/tree) + gall + time + gall * time

model2: total_herbivory ~ (1 | site/tree) + (gall | site) + gall + time + gall * time

LRT

|  | Df | AIC | BIC | logLik | Chisq | Chi Df | Pr(>Chisq) |
| --- | --- | --- | --- | --- | --- | --- | --- |
| model1 | 9 | -228.69 | -201.6 | 123.35 |  |  |  |
| model2 | 12 | -222.36 | -186.23 | 123.18 | 0 | 3 | 1 |

Herbivory chewers

model1: chewers ~ (1 | site/tree) + gall + time + gall * time

model2: chewers ~ (1 | site/tree) + (gall | site) + gall + time + gall * time

LRT

|  | Df | AIC | BIC | logLik | Chisq | Chi Df | Pr(>Chisq) |
| --- | --- | --- | --- | --- | --- | --- | --- |
| model1 | 9 | -204.65 | -177.55 | 111.32 |  |  |  |
| model2 | 12 | -197.53 | -161.4 | 110.76 | 0 | 3 | 1 |

Herbivory sceletonizers

models1: sceletonizers ~ (1 | site/tree) + gall + time + gall * time

models2: sceletonizers ~ (1 | site/tree) + (gall | site) + gall + time + gall * time

LRT

|  | Df | AIC | BIC | logLik | Chisq | Chi Df | Pr(>Chisq) |
| --- | --- | --- | --- | --- | --- | --- | --- |
| model1 | 9 | -466.18 | -439.08 | 242.09 |  |  |  |
| model2 | 12 | -460.24 | -424.11 | 242.12 | 0.0606 | 3 | 0.9961 |

Decomposition

model1: remaining.leaf.litter.mass.. ~ (1 | site) + (1 | block) + gall + time + gall * time

model2: remaining.leaf.litter.mass.. ~ (1 | site) + (1 | block) + (gall | site) + gall + time + gall * time

LRT

|  | Df | AIC | BIC | logLik | Chisq | Chi Df | Pr(>Chisq) |
| --- | --- | --- | --- | --- | --- | --- | --- |
| model1 | 7 | 560.32 | 578.34 | -273.16 |  |  |  |
| model2 | 10 | 564.25 | 589.99 | -272.12 | 2.07 | 3 | 0.5576 |

**Variation among trees within sites**

Phenol content

model1: phenols ~ (1 | site/tree) + gall + time + gall * time

model2: phenols ~ (1 | site/tree) + (gall| site/tree) + gall + time + gall * time

LRT

|  | Df | AIC | BIC | logLik | Chisq | Chi Df | Pr(>Chisq) |
| --- | --- | --- | --- | --- | --- | --- | --- |
| model1 | 7 | 939.4 | 957.63 | -462.7 |  |  |  |
| model2 | 13 | 950.87 | 984.74 | -462.44 | 0.523 | 6 | 0.9975 |

Chlorophyll content

model1: chlorophyll ~ (1 | site/tree) + gall + time + gall * time

model2: chlorophyll ~ (1 | site/tree) + (gall | site/tree) + gall + time + gall * time

LRT

|  | Df | AIC | BIC | logLik | Chisq | Chi Df | Pr(>Chisq) |
| --- | --- | --- | --- | --- | --- | --- | --- |
| model1 | 7 | 160.19 | 178.42 | -73.094 |  |  |  |
| model2 | 13 | 169.1 | 202.97 | -71.551 | 3.09 | 6 | 0.7979 |

CN

model1: CN ~ (1 | site/tree) + gall + time + gall * time

model2: CN ~ (1 | site/tree) + (gall | site/tree) + gall + time + gall * time

LRT

|  | Df | AIC | BIC | logLik | Chisq | Chi Df | Pr(>Chisq) |
| --- | --- | --- | --- | --- | --- | --- | --- |
| model1 | 7 | 600.26 | 618.5 | -293.13 |  |  |  |
| model2 | 13 | 610.16 | 644.03 | -292.08 | 2.11 | 6 | 0.9097 |

Total herbivory

model1: total_herbivory ~ (1 | site/tree) + gall + time + gall * time

model2: total_herbivory ~ (1 | site/tree) + (gall | site/tree) + gall + time + gall * time

LRT

|  | Df | AIC | BIC | logLik | Chisq | Chi Df | Pr(>Chisq) |
| --- | --- | --- | --- | --- | --- | --- | --- |
| model1 | 9 | -228.69 | -201.6 | 123.35 |  |  |  |
| model2 | 15 | -216.69 | -171.53 | 123.35 | 0 | 6 | 1 |

Herbivory chewer

model1: chewer ~ (1 | site/tree) + gall + time + gall * time

model2: chewer ~ (1 | site/tree) + (gall | site/tree) + gall + time + gall * time

LRT

|  | Df | AIC | BIC | logLik | Chisq | Chi Df | Pr(>Chisq) |
| --- | --- | --- | --- | --- | --- | --- | --- |
| model1 | 9 | -204.65 | -177.55 | 111.32 |  |  |  |
| model2 | 15 | -193.98 | -148.82 | 111.99 | 1.33 | 6 | 0.97 |

Herbivory sceletonisers

model1: sceletonisers ~ (1 | site/tree) + gall + time + gall * time

model2: skeletonisers ~ (1 | site/tree) + (gall | site/tree) + gall + tim e+ gall * time

LRT

|  | Df | AIC | BIC | logLik | Chisq | Chi Df | Pr(>Chisq) |
| --- | --- | --- | --- | --- | --- | --- | --- |
| model1 | 9 | -466.18 | -439.08 | 242.09 |  |  |  |
| model2 | 15 | -454.47 | -409.31 | 242.23 | 0.292 | 6 | 0.9995 |
